# Supplementary figures and images for: Ablation of Ezh2 in neural crest cells leads to aberrant enteric nervous system development in mice
Source: PLoS One. 2018 Aug 31;13(8):e0203391. doi: 10.1371/journal.pone.0203391 (PMC6118393; doi:10.1371/journal.pone.0203391)

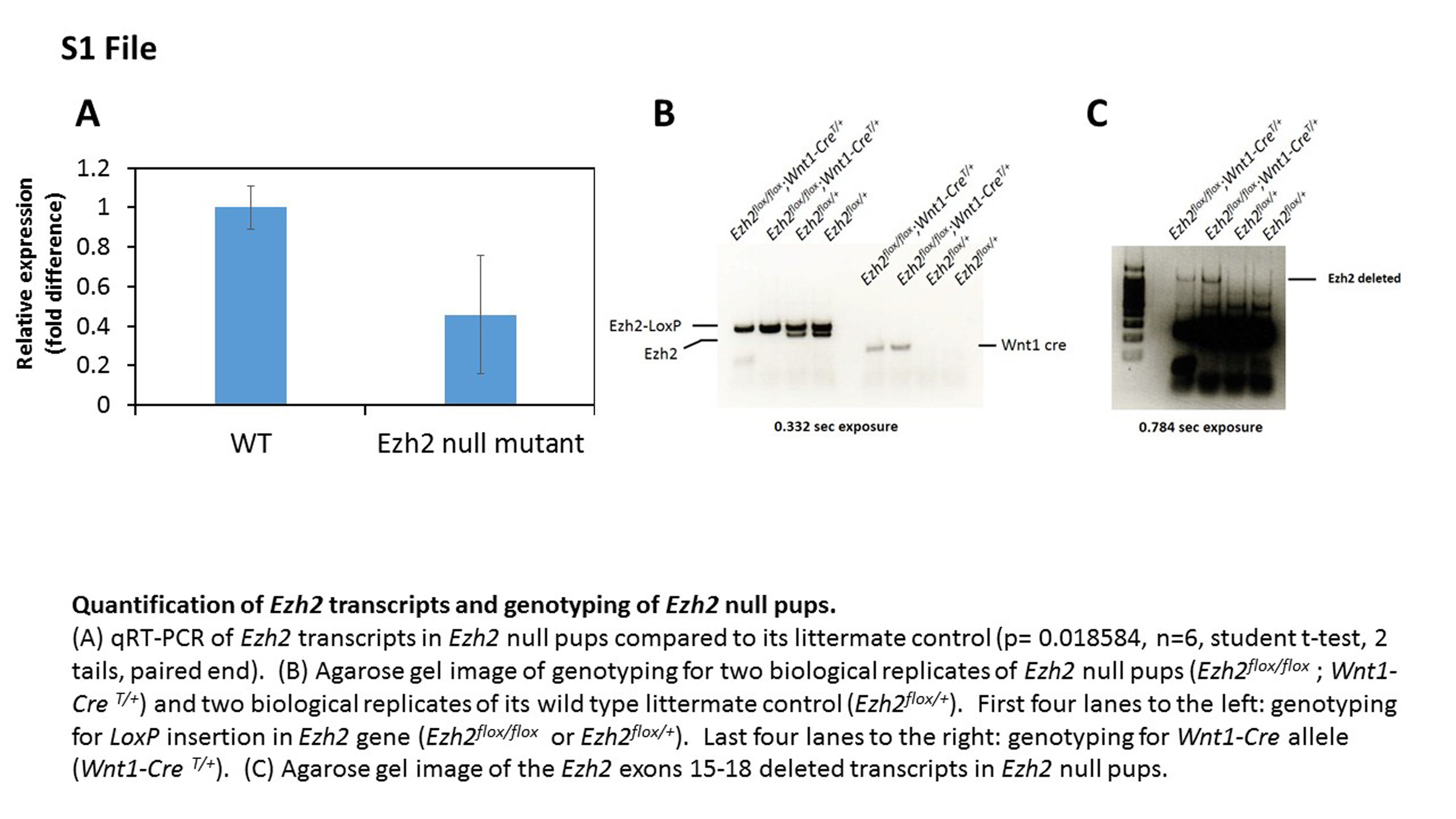

Supplement: S1 Fig — This file contains qRT-PCR results of Ezh2 transcripts and agarose gel electrophoresis images of the Ezh2 genotyping. (JPG) [file pone.0203391.s001.jpg]

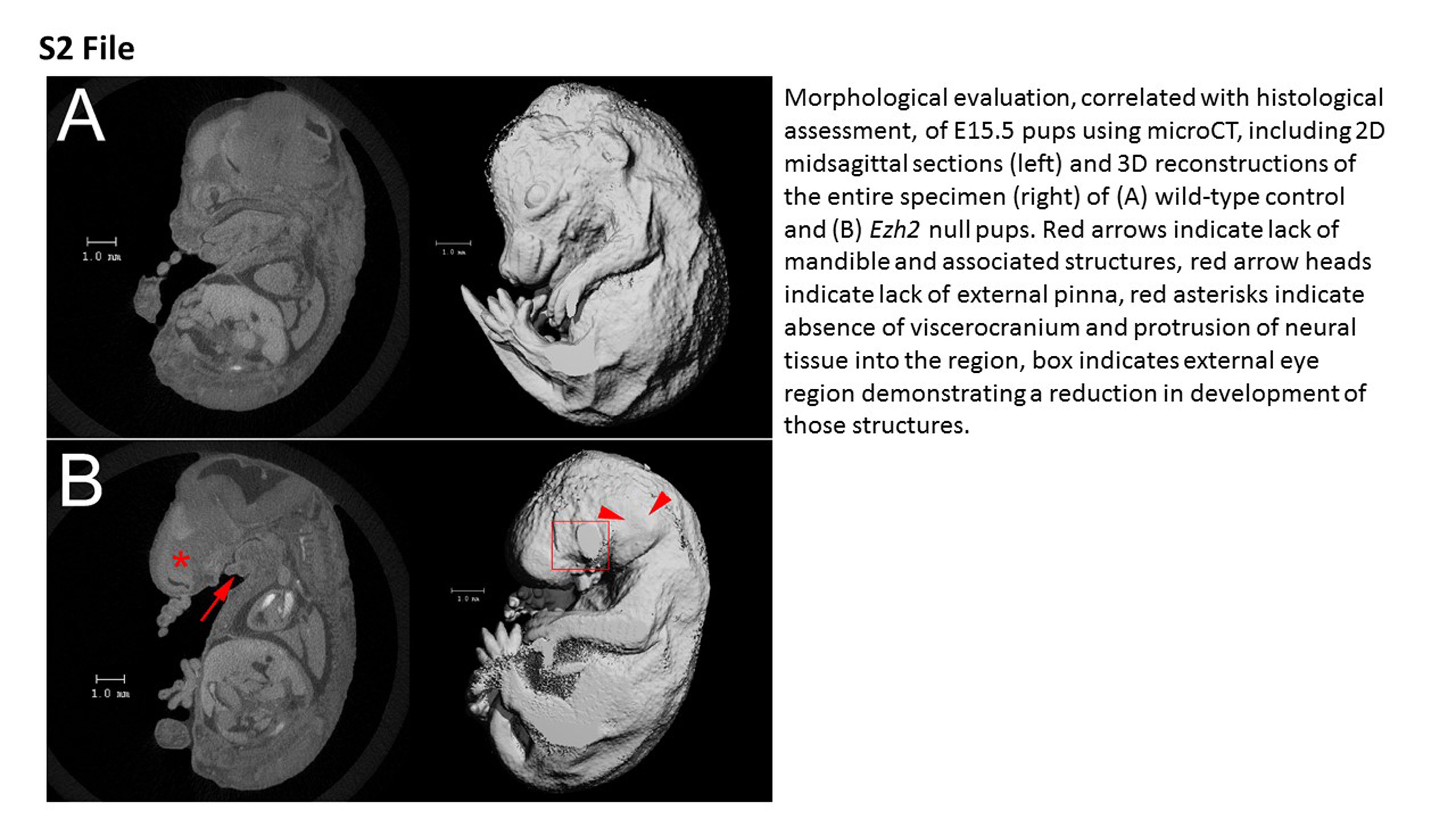

Supplement: S2 Fig — This file contains a set of images showing 2D and 3D microCT images of wild type and Ezh2 null pups. (JPG) [file pone.0203391.s002.jpg]

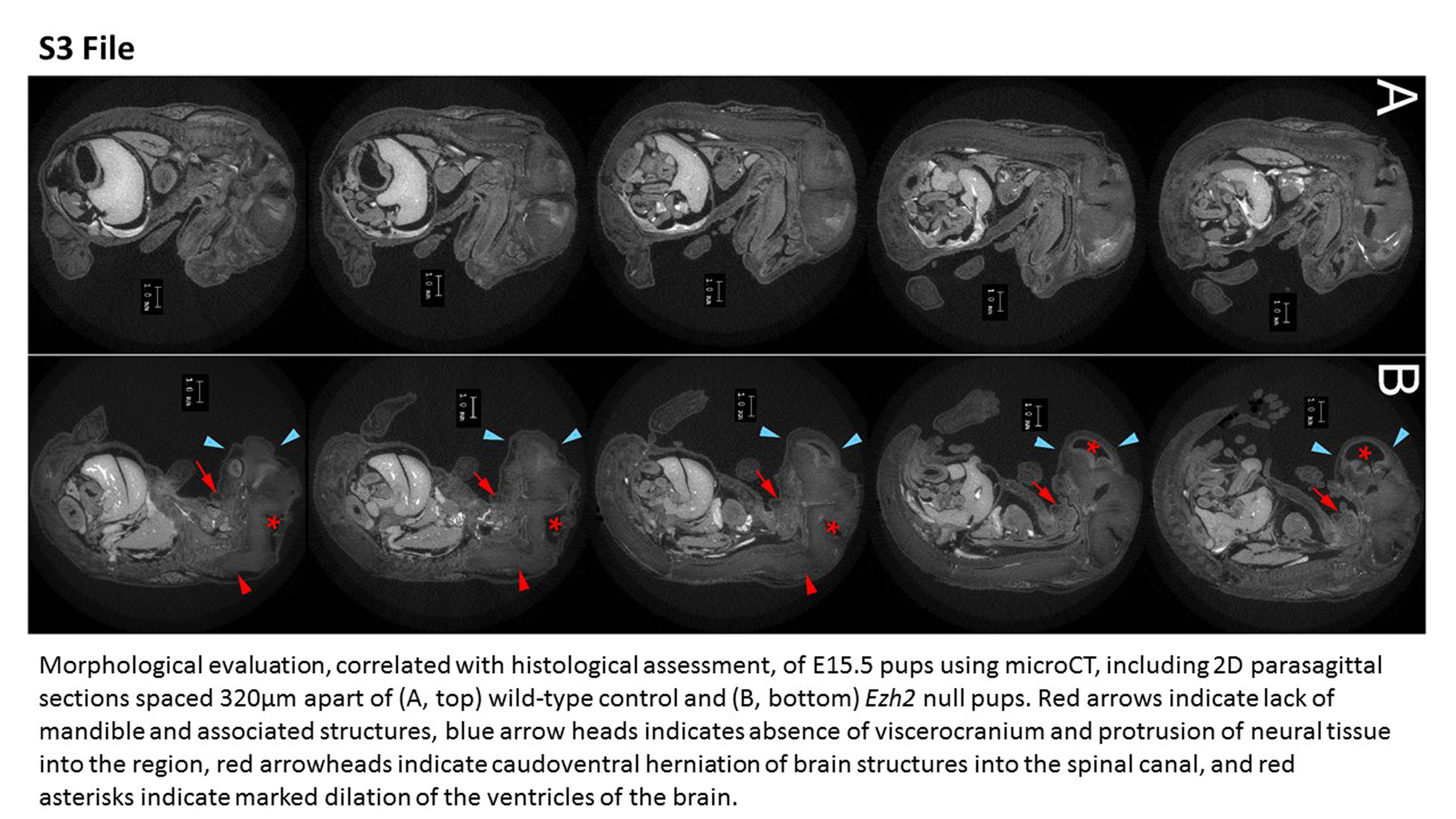

Supplement: S3 Fig — This file contains a set of images of wild type and Ezh2 null E15.5 embryos using micro CT. (JPG) [file pone.0203391.s003.jpg]

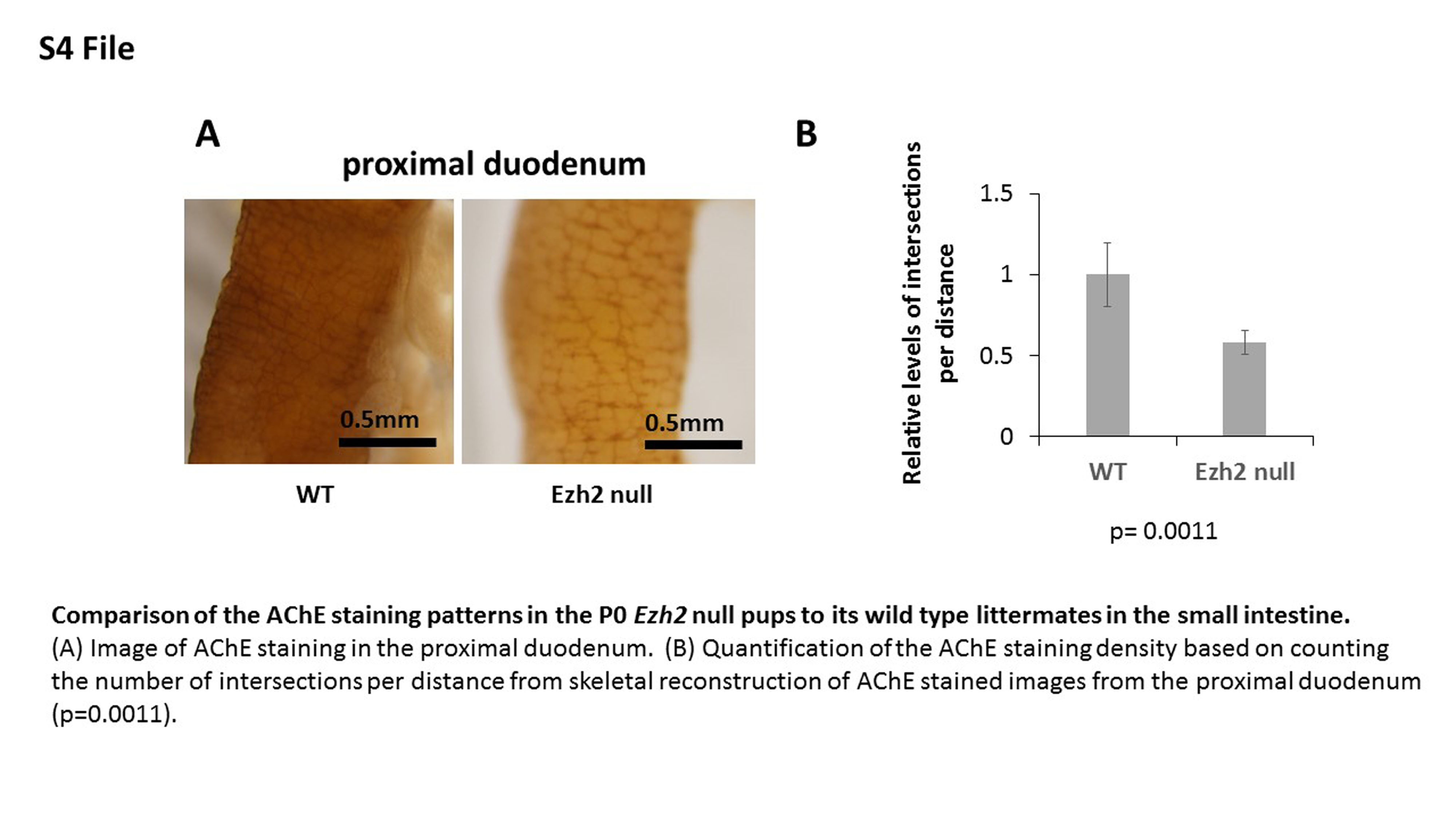

Supplement: S4 Fig — This file contains images of AChE stained small intestine in wild type and Ezh2 null pups. (JPG) [file pone.0203391.s004.jpg]

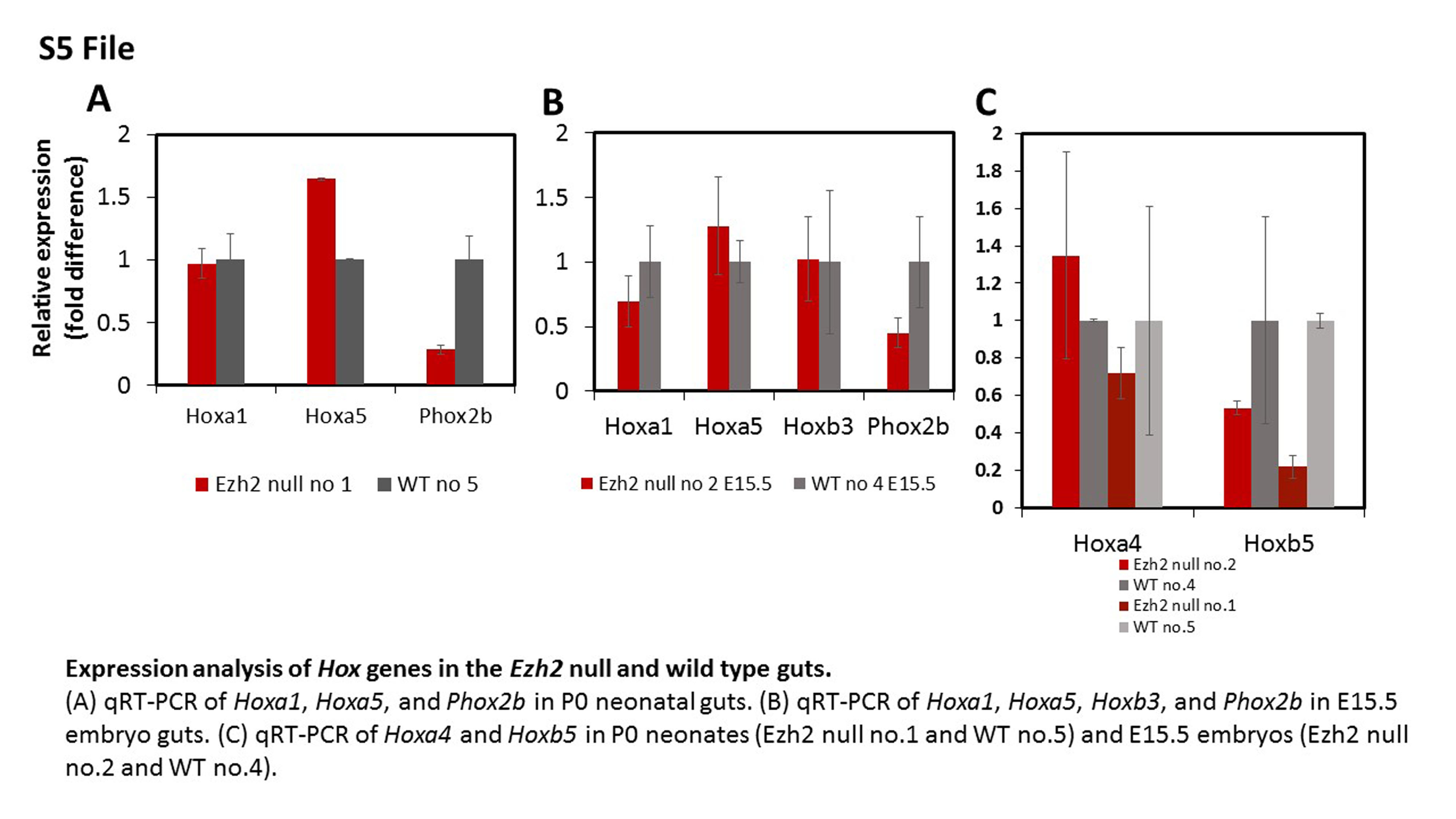

Supplement: S5 Fig — This file contains qRT-PCR results of Hox genes in the gastrointestinal tracts of Ezh2 null mice. (JPG) [file pone.0203391.s005.jpg]

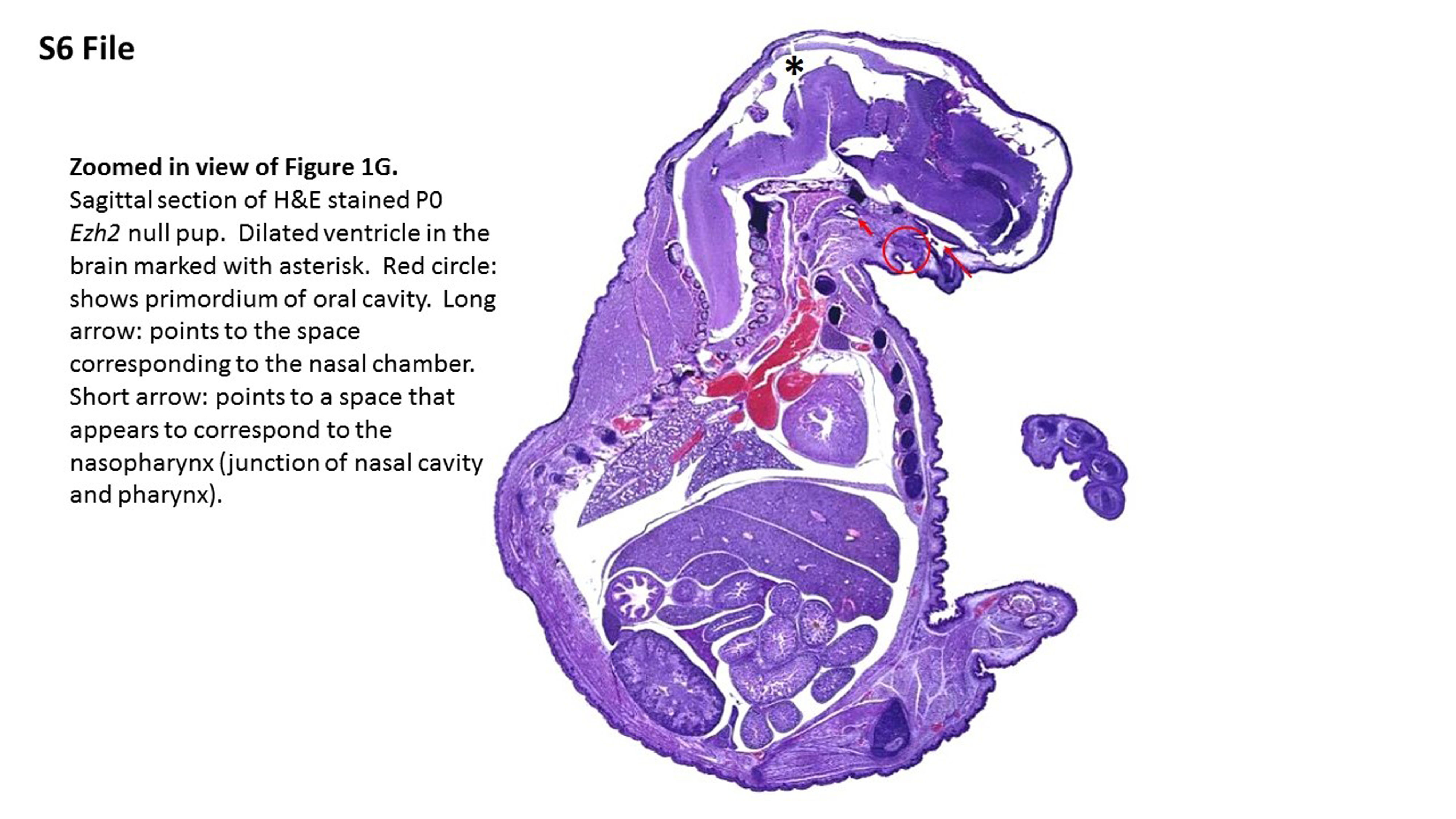

Supplement: S6 Fig — This file contains a zoomed in view of H&E staining presented in Fig 1G. (JPG) [file pone.0203391.s006.jpg]

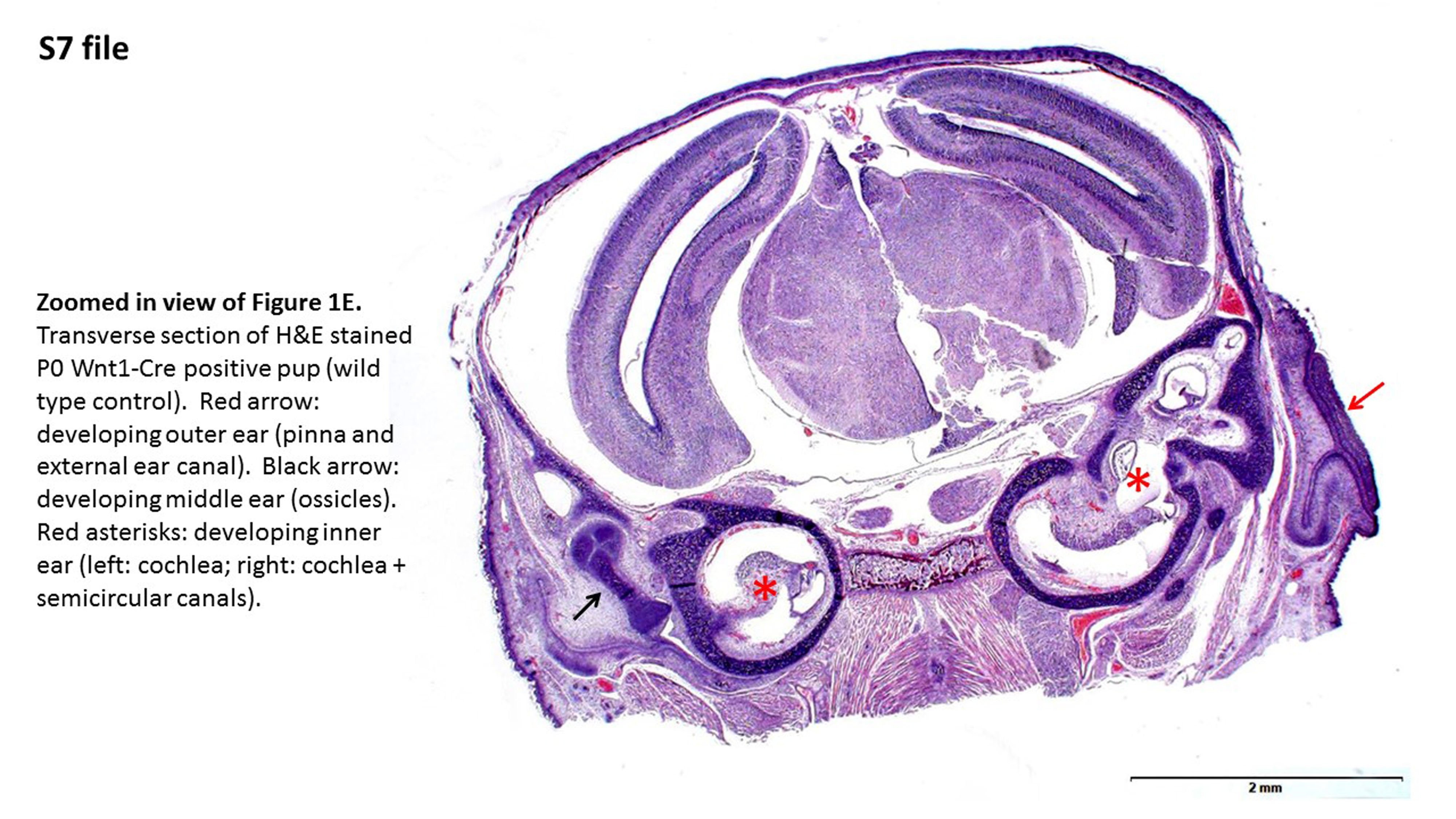

Supplement: S7 Fig — This file contains a zoomed in view of H&E staining presented in Fig 1E. (JPG) [file pone.0203391.s007.jpg]

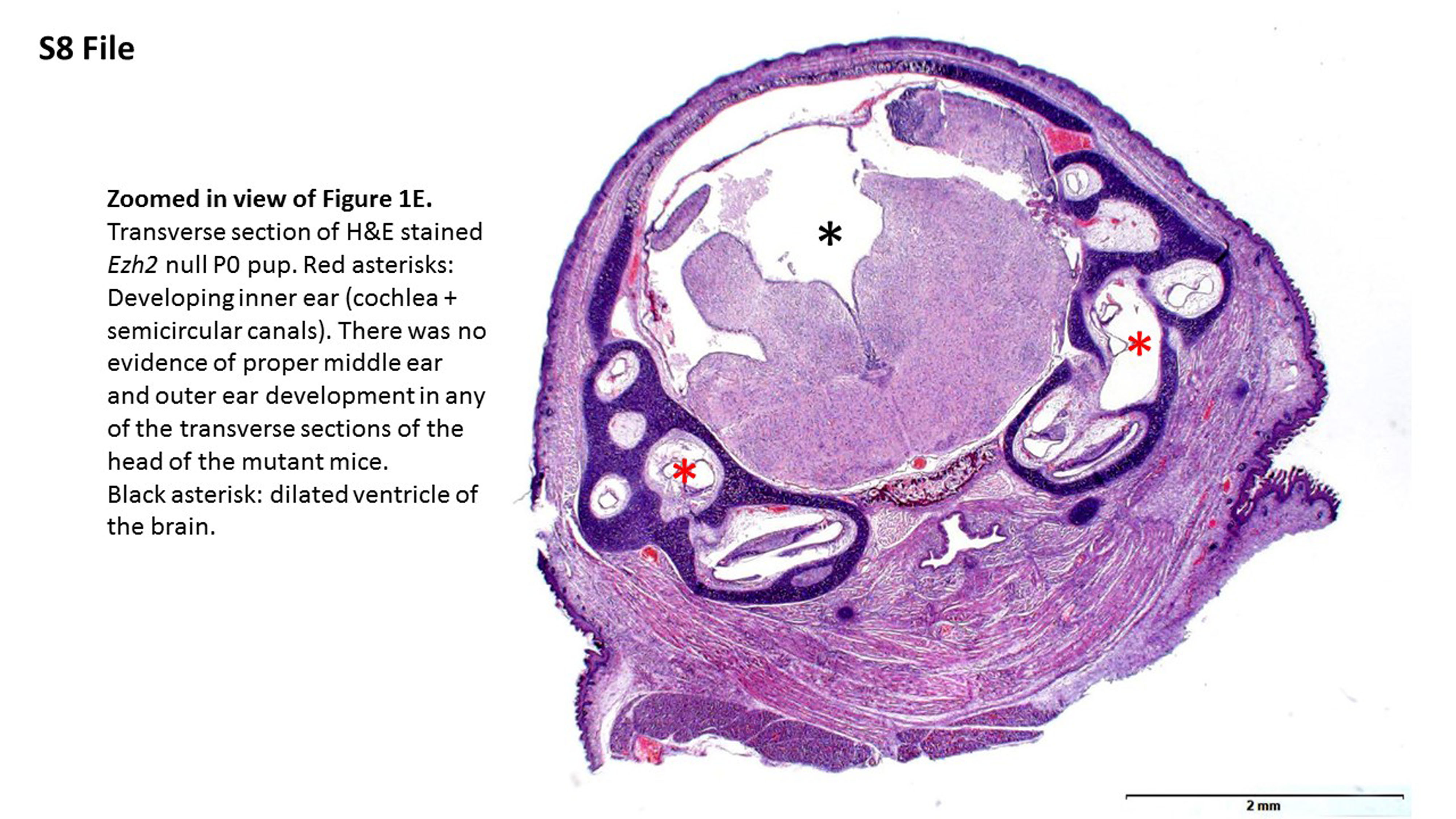

Supplement: S8 Fig — This file contains a zoomed in view of H&E staining presented in Fig 1E. (JPG) [file pone.0203391.s008.jpg]

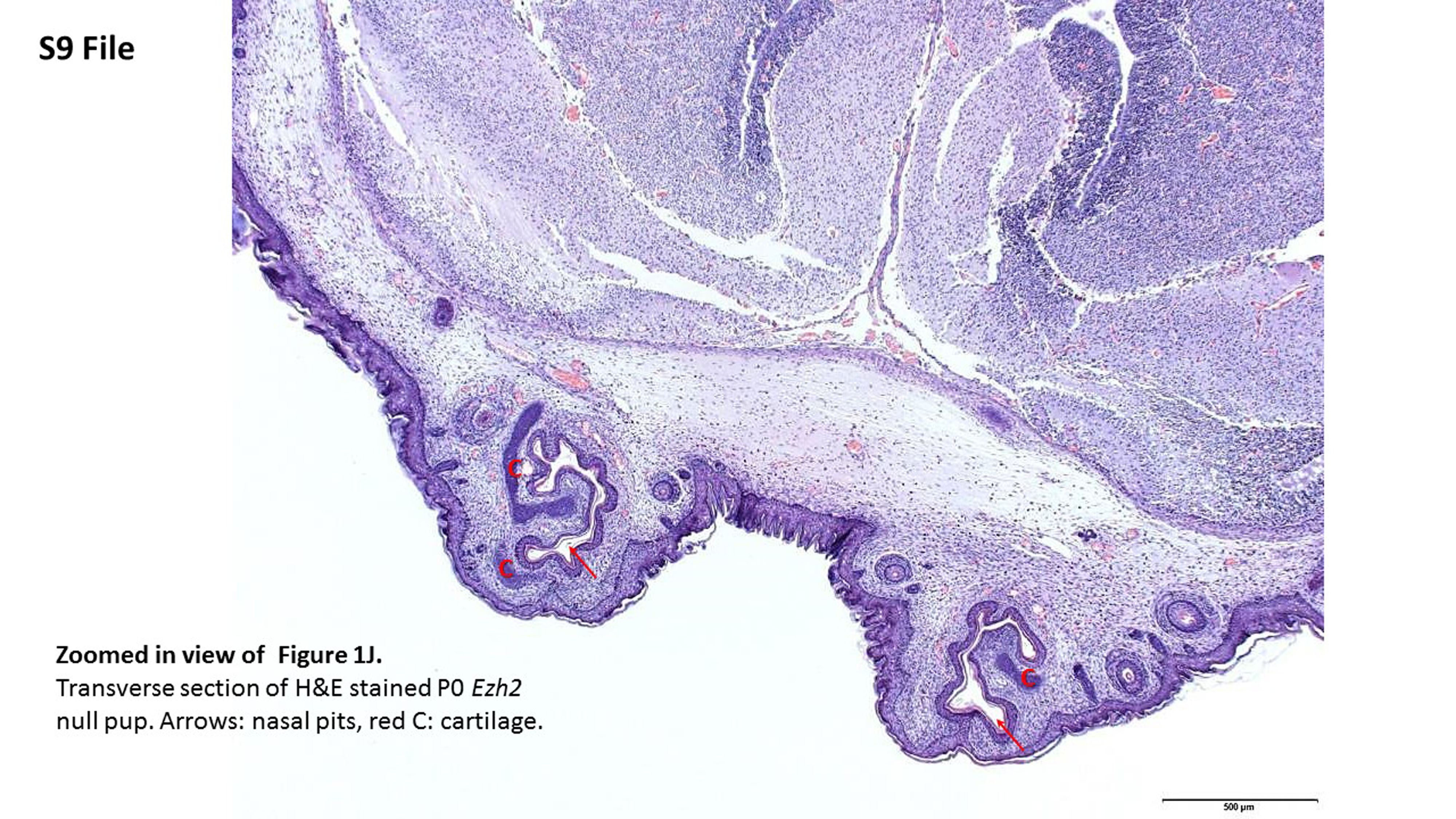

Supplement: S9 Fig — This file contains a zoomed in view of H&E staining presented in Fig 1J. (JPG) [file pone.0203391.s009.jpg]

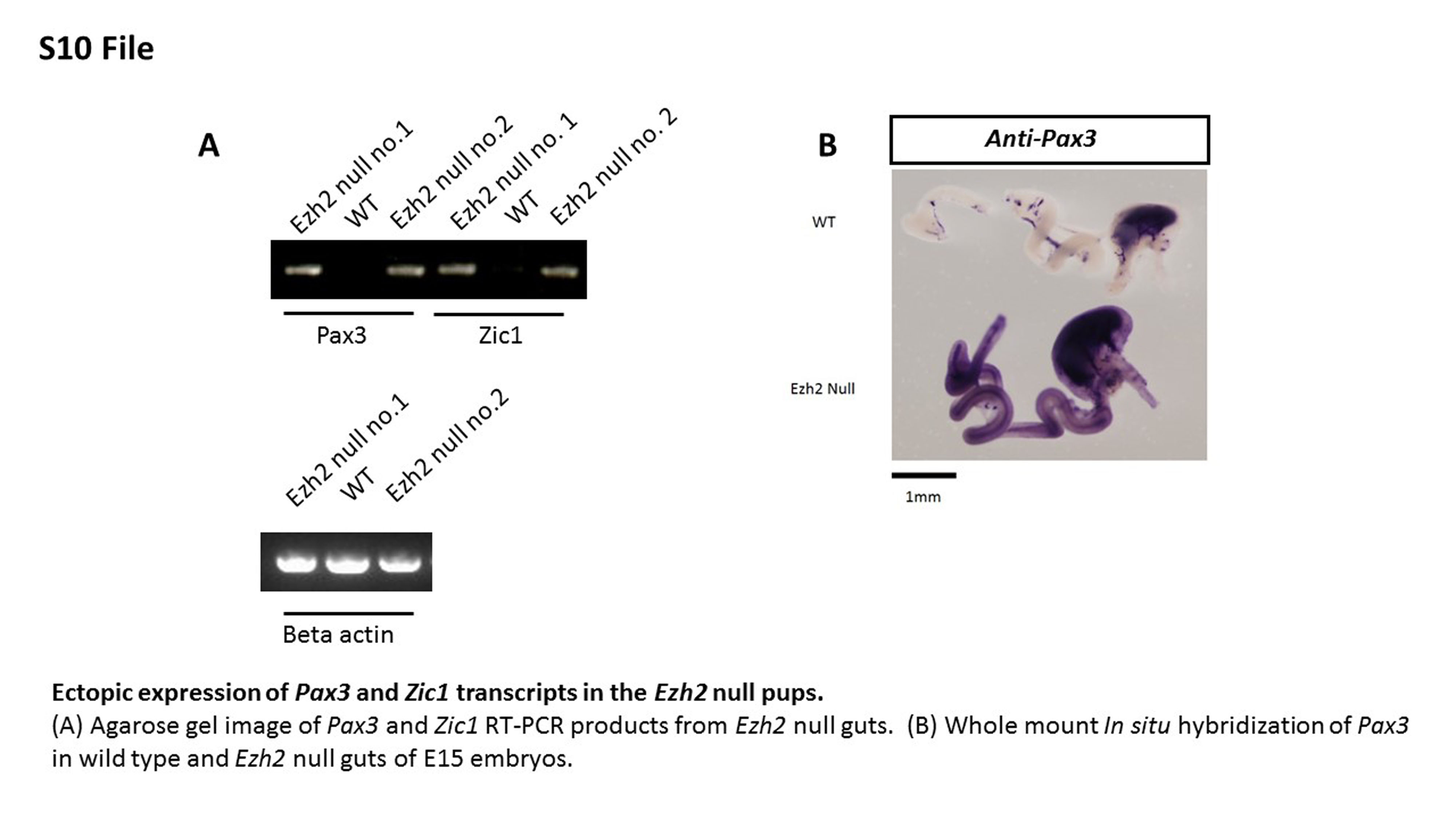

Supplement: S10 Fig — This file contains agarose gel images of Pax3 and Zic1 RT-PCR products from Ezh2 null guts, and whole mount in situ hybridization of Pax3 in wild type and Ezh2 null guts of E15 embryos. (JPG) [file pone.0203391.s010.jpg]

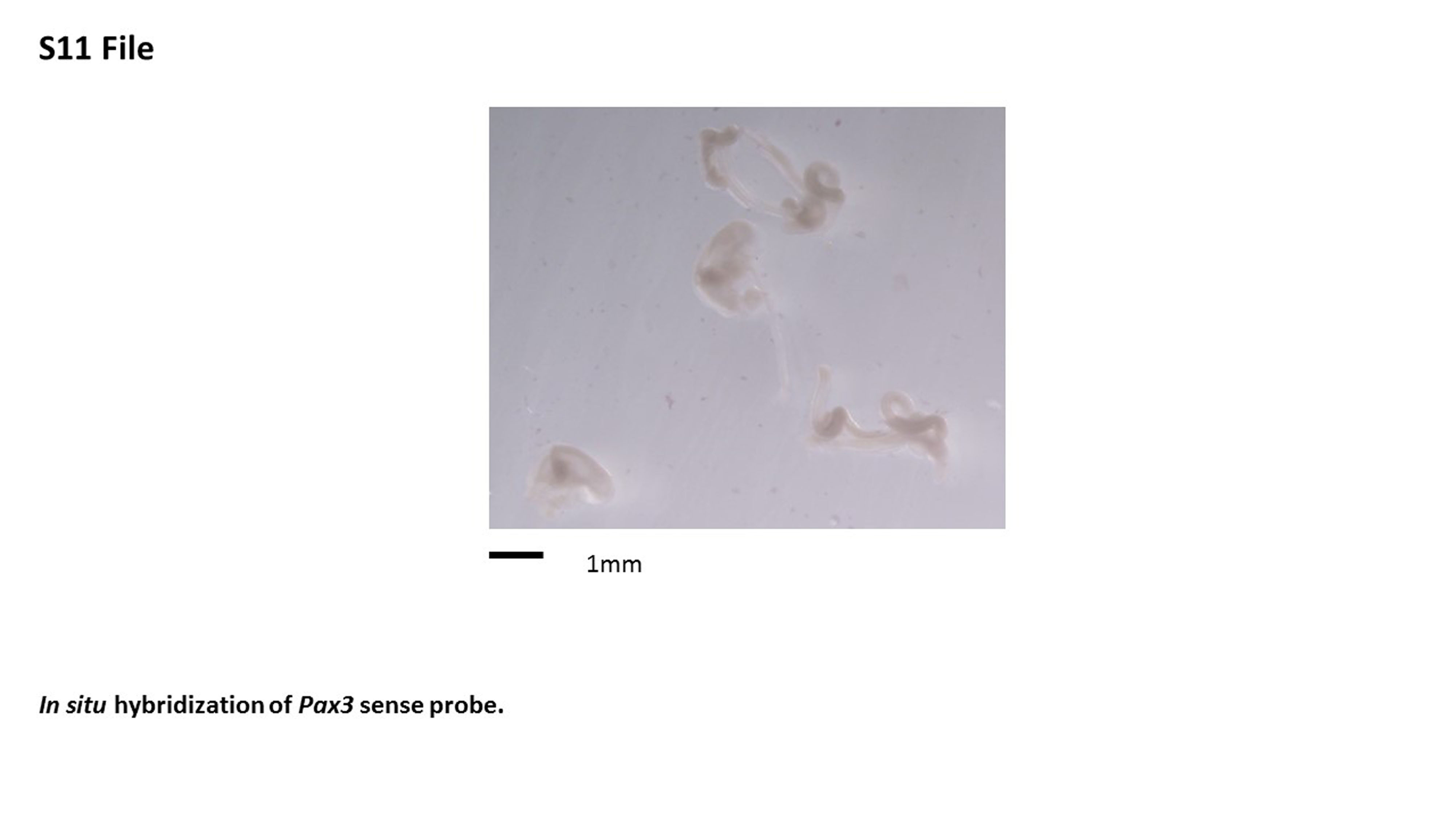

Supplement: S11 Fig — This file contains an image of in situ hybridization with Pax3 sense probe. (JPG) [file pone.0203391.s011.jpg]

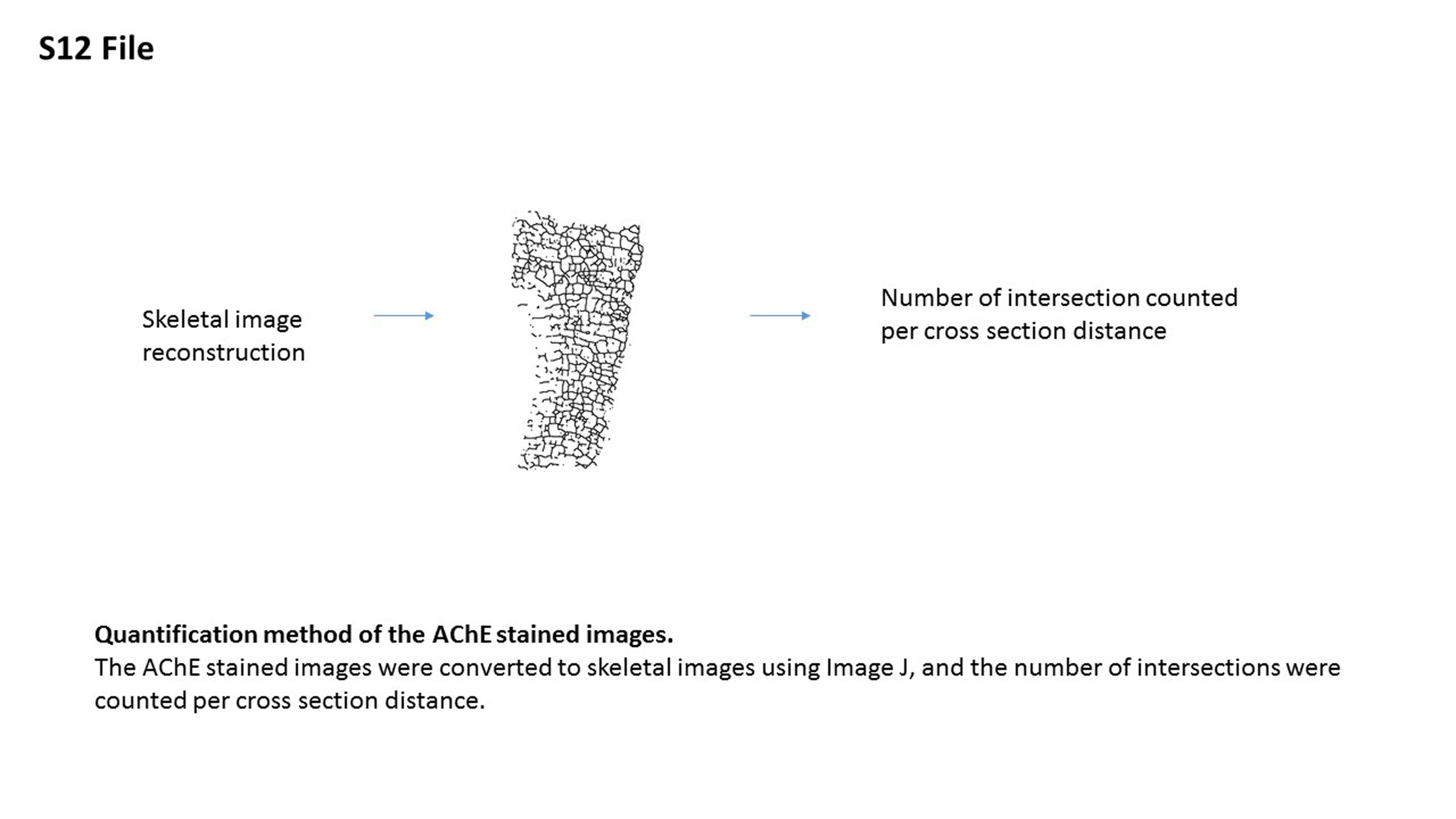

Supplement: S12 Fig — This file contains an image of an overview for quantifying the AChE stained images. (JPG) [file pone.0203391.s012.jpg]

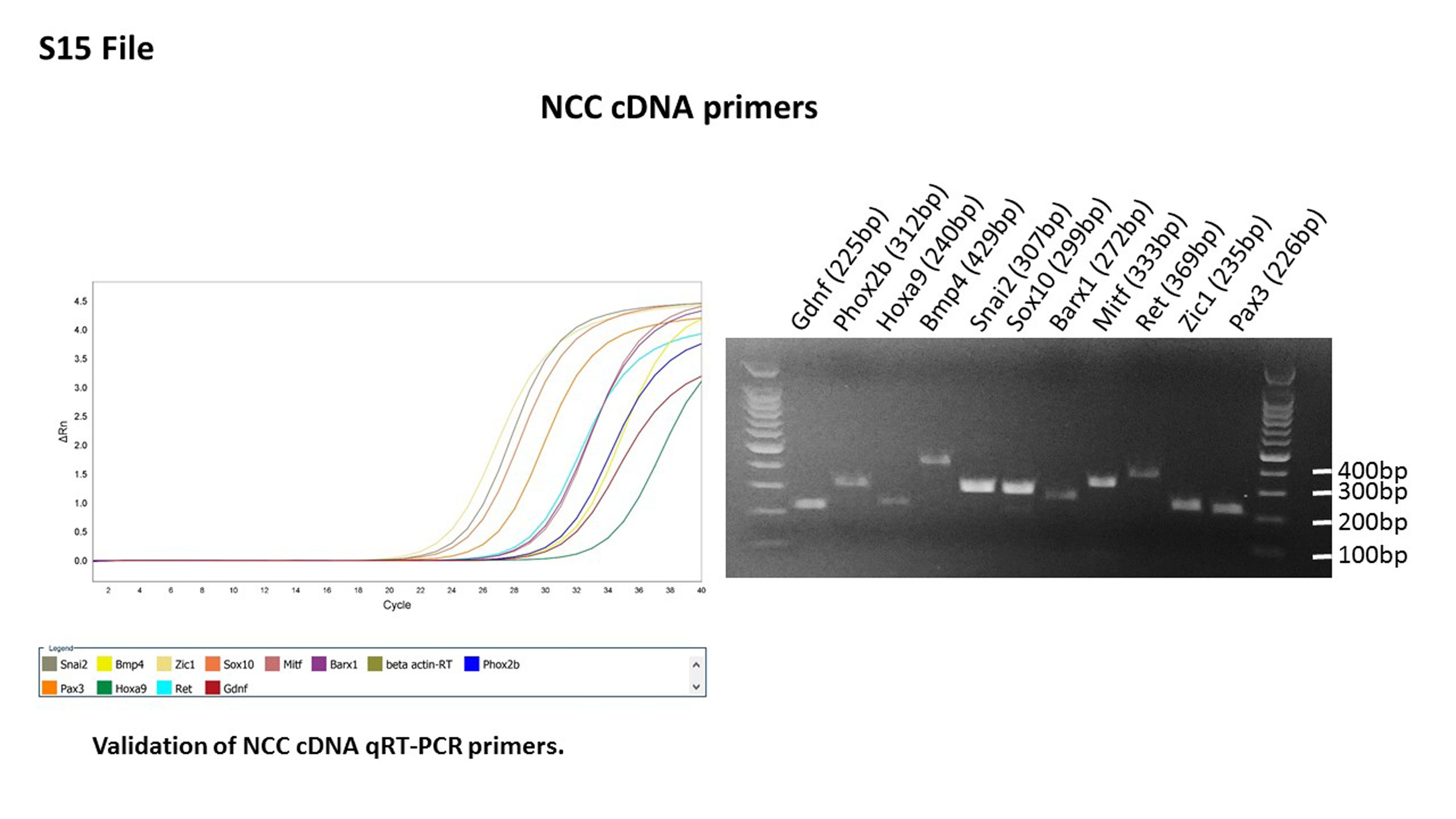

Supplement: S15 Fig — This file contains agarose gel electrophoresis image of all NCC cDNA primers used in this study. (JPG) [file pone.0203391.s015.jpg]

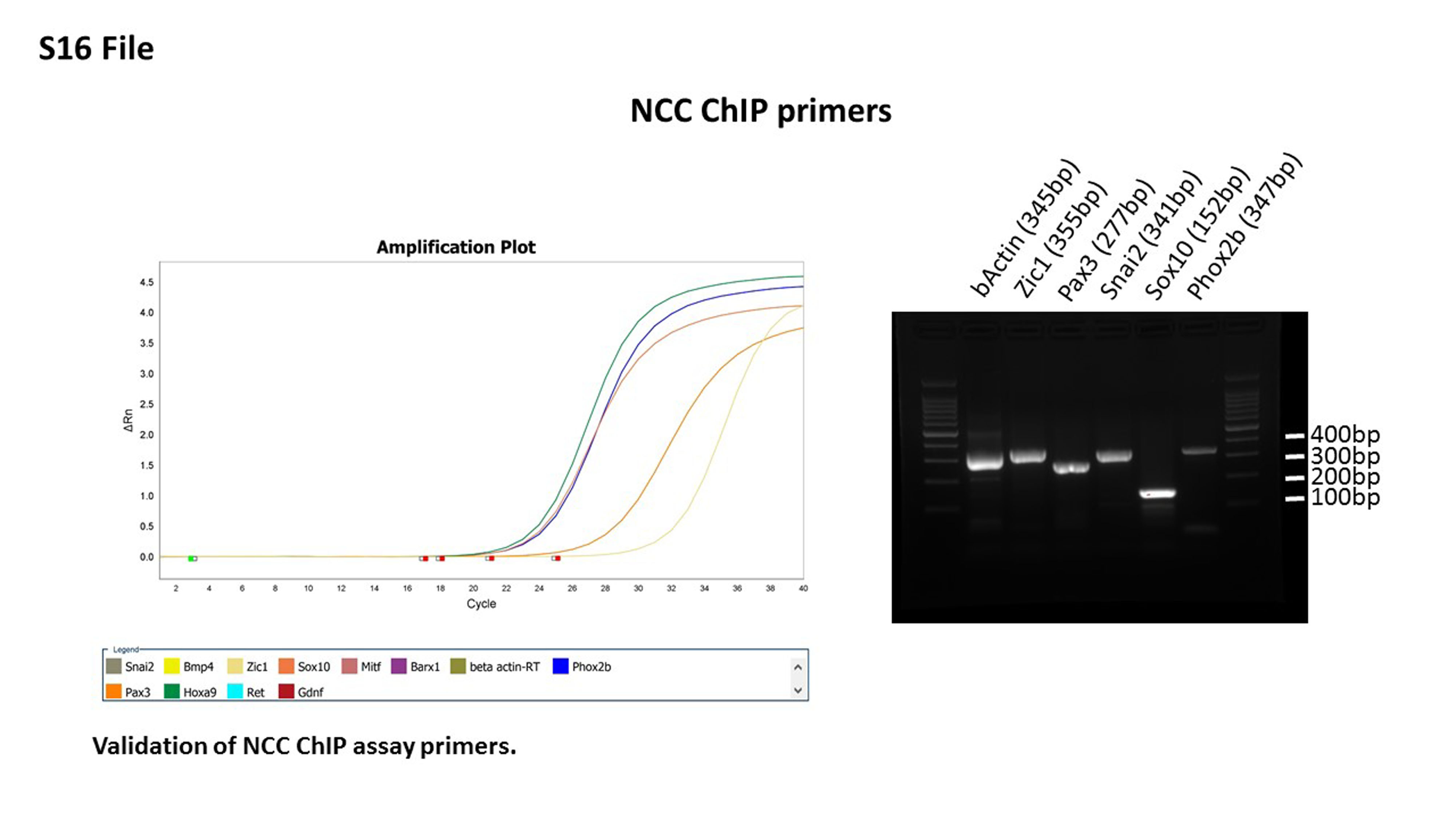

Supplement: S16 Fig — This file contains agarose gel electrophoresis images of all NCC ChIP assay primers used in this study. (JPG) [file pone.0203391.s016.jpg]

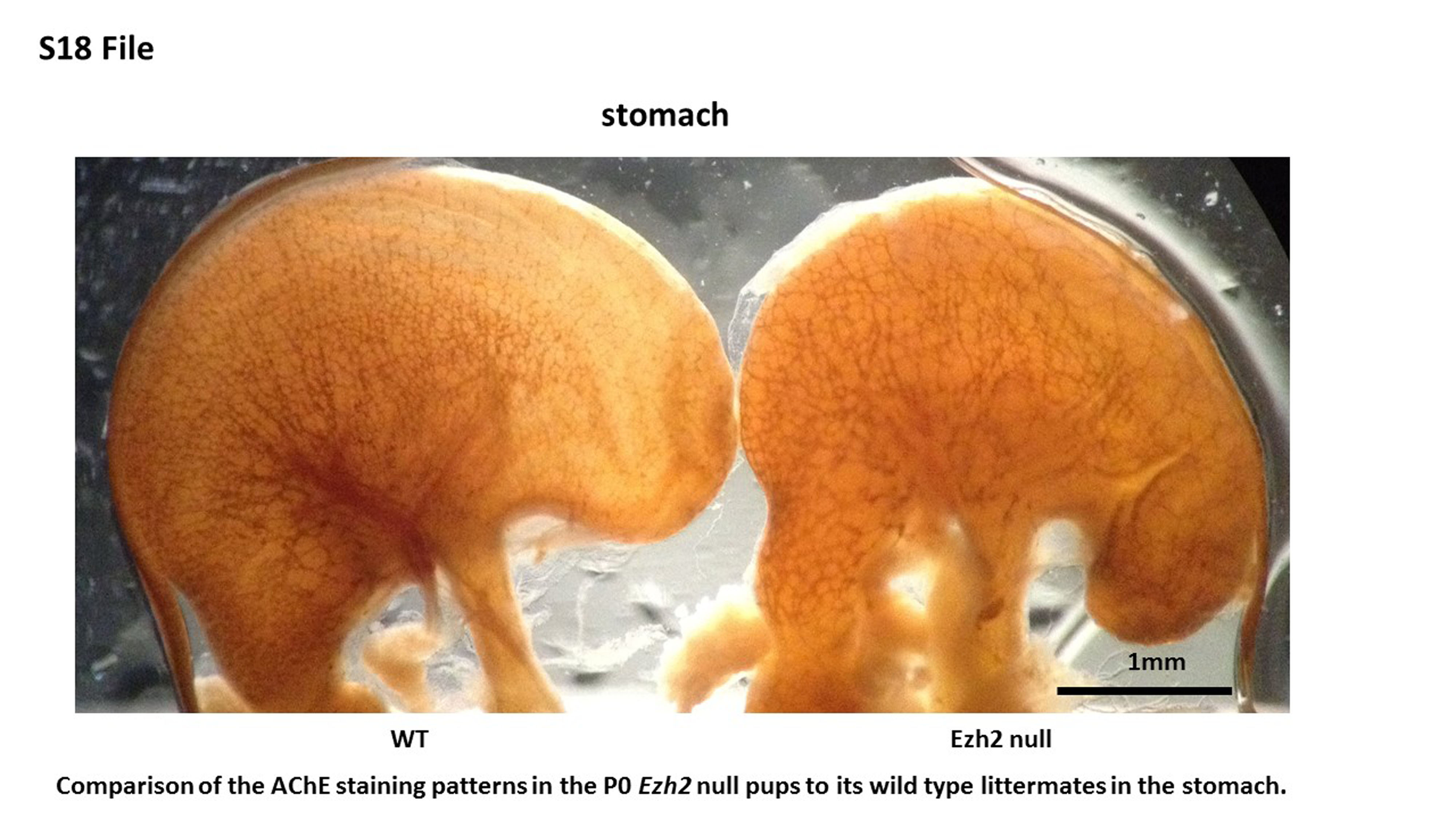

Supplement: S17 Fig — This file contains an image of wild type and Ezh2 null P0 stomachs. (JPG) [file pone.0203391.s017.jpg]

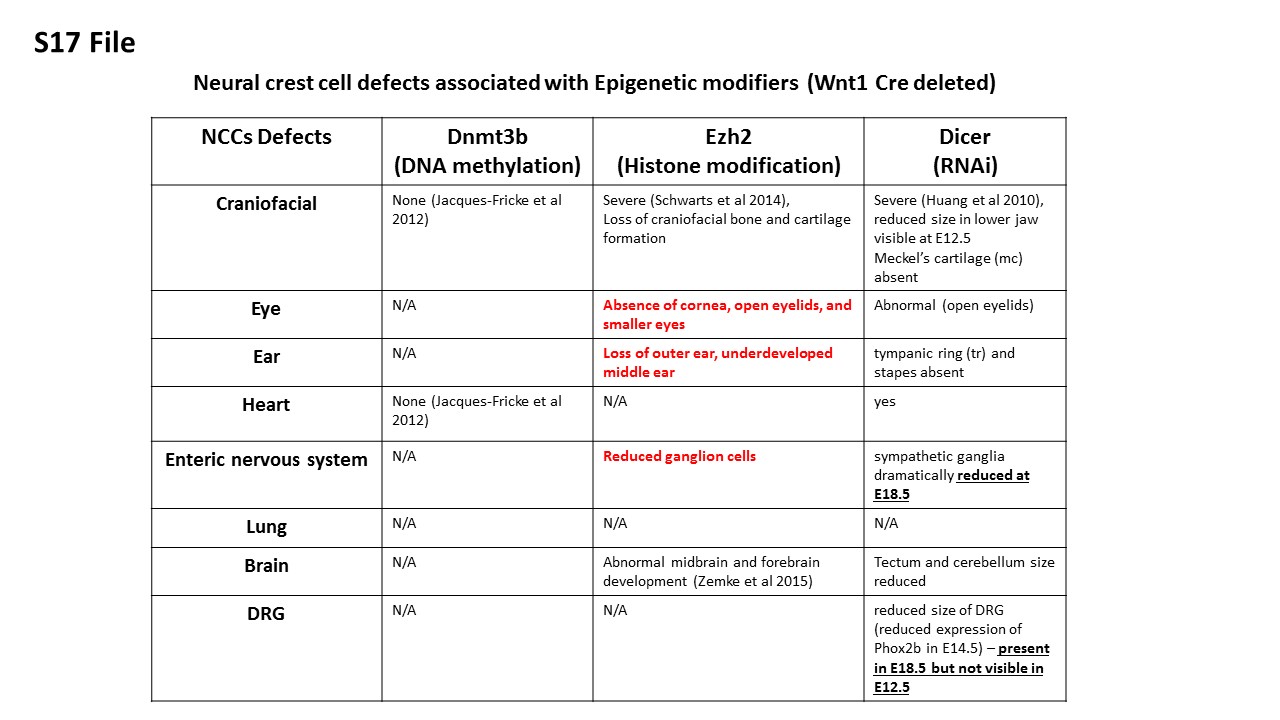

Supplement: S1 Table — This file contains a table summarizing the results of epigenetic modifiers that were deleted with Wnt1-Cre. The phenotypes reported in this current study are marked in red. (JPG) [file pone.0203391.s018.jpg]
