# Supplementary material for: Ablation of Ezh2 in neural crest cells leads to aberrant enteric nervous system development in mice
Source: PLoS One. 2018 Aug 31;13(8):e0203391. doi: 10.1371/journal.pone.0203391 (PMC6118393; doi:10.1371/journal.pone.0203391)
Supplement: S13 Fig — This file contains the primer sequences for NCC qRT-PCR. (DOCX) [file pone.0203391.s013.docx]

**S13 File. Primer sequences for NCC qRT-PCR**

GDNF-RT-A (225bp)

GCCGGTAAGAGGCTTCTCGAAG

GDNF-RT-B

GGCAGCTGCAGCCTGCCGATTC

PHOX2B-RT-A (312bp)

CCAGTGGCTTCCAGTATAAC

PHOX2B-RT-B

GGACTCTCGCCTCGGTGAGGT

HOXA9-RT-A (240bp)

ACTGACTATGCTTGTGGTTCT

HOXA9-RT-B

GTCCCGTGTGAGGTACATGTTA

BMP4-RT-A (429bp)

CACTTCTACAGATGTTTGGGCTG

BMP4-RT-B

TGTCCAGTAGTCGTGTGATGA

SNAI2-RT-A (307bp)

GAAGCCCAACTACAGCGAACTG

SNAI2-RT-B

CTTGGGCTGCAGTCTCTCCTCT

SOX10-RT-A (299bp)

GAGCTCAGCAAGACACTAGGCAAG

SOX10-RT-B

GAGGGGTGCTCTGGGTTCCCATCTG

BARX1-RT-A (272bp)

AGTCGCACCGTATTCACTGAG

BARX1-RT-B

TTGGCACGCTCTTGCTCCGT

MITF-RT-A (333bp)

GGGAACCATTCTCAAGGCCTCTG

MITF-RT-B

AGGTTGTTGGTAAAGGTGATGGT

RET-RT-A (369bp)

GGCACACCTCTGCTCTATGTC

RET-RT-B

CTCTGGGATGCAGAGATCCTG

PAX3-RT-A (226bp)

GCAGAATTACCCACGCAGCGGCT

PAX3-RT-B

CTCCTGGTACCTGCACAGGATCT

ZIC1-RT-A (235bp)

TCAACCACATCCGAGTGCACACAG

ZIC1-RT-B

TCGCACATCTTGCAAAGGTAGG
