# Supplementary material for: Ablation of Ezh2 in neural crest cells leads to aberrant enteric nervous system development in mice
Source: PLoS One. 2018 Aug 31;13(8):e0203391. doi: 10.1371/journal.pone.0203391 (PMC6118393; doi:10.1371/journal.pone.0203391)
Supplement: S14 Fig — This file contains the primer sequences for NCC ChIP assays. (DOCX) [file pone.0203391.s014.docx]

**S14 File. Primer sequences for NCC ChIP assays**

bACTIN-1A (335bp)

GACATGGAGAAGATCTGGCA

bACTIN-1B

CTTCATGAGGTAGTCCGTCA

ZIC1-A (355bp)

CCATGAACGTGAACATGGCTG

ZIC1-B

TCTGAACGCGCGAAAACCTTG

PAX3-A (277bp)

CTCGGTGTCACGACGGGAGGAGA

PAX3-B

CTCCGGATCTCGGAGAGCTCCT

HOXA9-A (341bp)

GTATGGAAAGCTCAGACAGCAAA

HOXA9-B

TCCACCTTTCTCTCGACAGCAC

SOX10-A (152bp)

AGCTGGACCGCACACCTTGGGACA

SOX10-B

CTCTCCCATCGCAAAGCAGGGGTGA

PHOX2B-A (347bp)

CTCTAGAGACCTCAGACAAGG

PHOX2B-B

CGTGGCCCCAAAAGTGGTCCT
